# Supplementary material for: An open-source framework for large-scale, flexible evaluation of biomedical text mining systems
Source: J Biomed Discov Collab. 2008 Jan 29;3:1. doi: 10.1186/1747-5333-3-1 (PMC2276192; doi:10.1186/1747-5333-3-1)
Supplement: Additional file 1 — Evaluation platform source code. The additional file contains the software evaluation framework discussed in this paper. The most current release can be downloaded from SourceForge.net [1]. See the accompanying README file for instructions on installation and use. Also included in the distribution is a collection of UIMA wrappers for some commonly used BioNLP tools and annotated corpora. [file 1747-5333-3-1-S1.zip › bionlp_uima_repository-1.0.1/docs/api/edu/uchsc/ccp/util/nlp/annotation/Annotation_Util.html]

Annotation\_Util (Center for Computational Pharmacology Util/UIMA API)


|  |  |  |
| --- | --- | --- |
| **Overview** \| **Package** \| **Class** \| **Tree** \| **Deprecated** \| **Index** \| **Help** | |  |
| PREV CLASS   **NEXT CLASS** | **FRAMES**    **NO FRAMES**     **All Classes** |  |
| SUMMARY: NESTED \| FIELD \| CONSTR \| METHOD | DETAIL: FIELD \| CONSTR \| METHOD |  |


---


## edu.uchsc.ccp.util.nlp.annotation Class Annotation\_Util

```
java.lang.Object
  edu.uchsc.ccp.util.nlp.annotation.Annotation_Util
```

---

``` public class Annotation_Util extends Object ```

**Author:**
:   williamb

---

**Constructor Summary**

`Annotation_Util()`


| **Method Summary** | |
| --- | --- |
| `static ClassMention` | `createTokenMention(String posLabel, String stem, String lemma, int tokenNumber)` |
| `static List<Object>` | `getSlotValuesByName(TextAnnotation ta, String slotName)` |
| `static void` | `swapAnnotationInfo(TextAnnotation fromTA, TextAnnotation toTA)` |

**Methods inherited from class java.lang.Object**

`clone, equals, finalize, getClass, hashCode, notify, notifyAll, toString, wait, wait, wait`

**Constructor Detail**

### Annotation\_Util

```
public Annotation_Util()
```


**Method Detail**

### createTokenMention

```
public static ClassMention createTokenMention(String posLabel,
                                              String stem,
                                              String lemma,
                                              int tokenNumber)
```

---


### swapAnnotationInfo

```
public static void swapAnnotationInfo(TextAnnotation fromTA,
                                      TextAnnotation toTA)
```

---


### getSlotValuesByName

```
public static List<Object> getSlotValuesByName(TextAnnotation ta,
                                               String slotName)
```


---


|  |  |  |
| --- | --- | --- |
| **Overview** \| **Package** \| **Class** \| **Tree** \| **Deprecated** \| **Index** \| **Help** | |  |
| PREV CLASS   **NEXT CLASS** | **FRAMES**    **NO FRAMES**     **All Classes** |  |
| SUMMARY: NESTED \| FIELD \| CONSTR \| METHOD | DETAIL: FIELD \| CONSTR \| METHOD |  |


---
